# Melatonin Differentially Modulates Temozolomide Responses in Glioblastoma and Endothelial Cells: Associations with Global DNA Methylation and Cortistatin Levels

**DOI:** 10.3390/cimb48100984

**Published:** 2026-09-24

**Authors:** Gul Ipek Gundogan, Asiye Gok Yurttas, Yasemin Musteri Oltulu, Enver Ciraci, Halil Ibrahim Arslan, Tugba Elgun

**Affiliations:** 1 Department of Histology and Embryology, Faculty of Medicine, Biruni University, 34015 Istanbul, Türkiye; ggundogan@biruni.edu.tr; 2 Biruni Advanced Technology Application and Research Center (B@MER), Biruni University, 34015 Istanbul, Türkiye; yoltulu@biruni.edu.tr (Y.M.O.); eciraci@biruni.edu.tr (E.C.); halila@biruni.edu.tr (H.I.A.); telgun@biruni.edu.tr (T.E.); 3 Department of Biochemistry, Faculty of Medicine, Istanbul Atlas University, 34408 Istanbul, Türkiye; 4 Department of Medical Biology, Faculty of Medicine, Biruni University, 34015 Istanbul, Türkiye; 5 Department of Biochemistry, Faculty of Pharmacy, Biruni University, 34015 Istanbul, Türkiye

**Keywords:** glioblastoma, temozolomide, melatonin, U87MG cells, HUVECs, global DNA methylation, CORT, relative MTT metabolic activity, cell cycle

## Abstract

Glioblastoma (GBM) is the most aggressive primary brain tumor in adults and remains highly resistant to current therapeutic strategies. Temozolomide (TMZ) is a standard chemotherapeutic agent used in GBM treatment; however, intrinsic and acquired resistance limits its efficacy. This study investigated treatment-associated response patterns to TMZ and melatonin in U87MG glioblastoma cells and human umbilical vein endothelial cells (HUVECs). Relative MTT metabolic activity was assessed using the MTT assay, and cell-cycle distribution was analyzed by flow cytometry. Global DNA methylation was evaluated using a 5-methylcytosine assay, with calibration-derived values expressed as %5-mC. CORT was assessed semi-quantitatively by ELISA, with background-corrected OD values normalized to the corresponding untreated control within each experimental run and expressed as relative CORT levels (% of control). Linear mixed-effects models were used to evaluate TMZ concentration, melatonin exposure, and the TMZ × melatonin interaction, with biological experiment included as a random effect. TMZ and melatonin produced treatment-condition- and cell-type-associated response patterns. In HUVECs, relative MTT metabolic activity remained comparatively high across most TMZ concentrations, whereas a more pronounced decrease was observed at 100 µM TMZ during melatonin co-treatment. U87MG cells showed a non-linear MTT response across TMZ concentrations, and the magnitude and direction of the melatonin-associated effect varied according to TMZ dose. Significant TMZ × melatonin interactions were identified for relative MTT metabolic activity in both cell types. Global DNA methylation-related measurements also showed treatment-associated differences, with distinct response patterns between HUVEC and U87MG cells; calibration-derived %5-mC values were used for quantitative presentation of global DNA methylation. Relative CORT levels likewise varied across treatment conditions, with larger relative changes observed in U87MG cells under several combination conditions. The observed findings should be interpreted as exploratory and hypothesis-generating. The present data do not establish therapeutic selectivity, pharmacological synergy, a causal epigenetic mechanism, or a CORT-mediated stress-response pathway. Further validation using additional glioblastoma models, non-malignant neural controls, fully quantitative and appropriately normalized CORT measurements, and targeted functional pathway analyses is required before mechanistic or clinical conclusions can be drawn.

## 1. Introduction

Glioblastoma multiforme (GBM) is the most aggressive and lethal primary brain tumor in adults and is characterized by rapid proliferation, diffuse infiltration, and profound therapeutic resistance. Despite advances in neurosurgical techniques and multimodal treatment strategies, the prognosis for GBM patients remains poor, with a median overall survival of approximately 15 months following maximal surgical resection combined with radiotherapy and chemotherapy [1,2]. Temozolomide (TMZ), an oral alkylating agent, constitutes the backbone of current chemotherapeutic regimens for GBM and exerts its cytotoxic effects primarily through methylation of DNA at the O^6^ position of guanine, leading to DNA damage and tumor cell death [1,2,3]. However, intrinsic and acquired resistance to TMZ remains a major clinical challenge and is frequently associated with enhanced DNA repair capacity, epigenetic dysregulation, and activation of survival signaling pathways.

Recent molecular studies have highlighted that glioblastoma progression and therapeutic resistance are strongly influenced by epigenetic alterations, including aberrant DNA methylation patterns that contribute to tumor heterogeneity and genomic instability [4,5,6,7]. These epigenetic changes can influence gene expression programs that regulate tumor proliferation, therapy resistance, and cellular stress responses. Consequently, modulation of the epigenetic landscape has emerged as a promising strategy for improving therapeutic responses in glioblastoma.

Melatonin (N-acetyl-5-methoxytryptamine) is an endogenous indoleamine best known for its role in circadian rhythm regulation and redox homeostasis. In recent years, melatonin has gained considerable attention as a pleiotropic molecule with multiple oncostatic properties, including inhibition of tumor cell proliferation, modulation of cell cycle progression, induction of apoptosis, and regulation of oxidative stress pathways [8,9,10]. Importantly, several studies have demonstrated that melatonin may exert differential biological effects in malignant and non-malignant cells, suggesting that it can selectively influence tumor cell signaling while preserving the functional stability of normal tissues [8,9,10].

Accumulating evidence further indicates that melatonin may enhance the therapeutic efficacy of TMZ in glioblastoma models. Experimental studies have shown that melatonin can sensitize glioblastoma cells to TMZ by modulating multiple signaling pathways associated with tumor survival, inflammation, and oxidative stress [11]. In addition, melatonin has been implicated in the regulation of epigenetic processes, including DNA methylation dynamics and chromatin remodeling, which play critical roles in tumor progression and therapy resistance [9,10,12].

Despite these promising observations, the context-dependent cellular responses to melatonin and TMZ remain incompletely understood, particularly when comparing malignant glioblastoma cells with non-malignant endothelial cells. Moreover, the potential contribution of epigenetic remodeling and stress-associated signaling molecules to the combined effects of melatonin and TMZ has not been fully characterized [11].

Therefore, the present study aimed to characterize the effects of melatonin, alone and in combination with temozolomide, on relative MTT metabolic activity, cell-cycle distribution, and global DNA methylation-related measurements in U87MG glioblastoma cells and human umbilical vein endothelial cells (HUVECs). In addition, CORT was evaluated as an exploratory treatment-associated marker. The study was designed to compare response patterns across cellular contexts rather than to establish tumor selectivity or a causal epigenetic or stress-response mechanism.

## 2. Materials and Methods

Human glioblastoma cell line U87MG (ATCC^®^ HTB-14™, American Type Culture Collection, Manassas, VA, USA) and human umbilical vein endothelial cells (HUVEC; PCS-100-010™ ATCC^®^, American Type Culture Collection, Manassas, VA, USA) were used in this study to investigate the differential effects of melatonin and temozolomide (TMZ) on cancerous and non-cancerous cells. U87MG cells were used between passages 8–10, and HUVECs were used between passages 16–20. U87MG cells were selected as a widely used in vitro model for glioblastoma due to their high proliferative capacity and well-characterized response to chemotherapeutic agents commonly used in glioblastoma treatment [3]. HUVECs were included as a representative model of normal endothelial cells to evaluate potential cytotoxic or protective effects of the treatments on healthy vascular endothelium and to provide insight into potential vascular responses associated with glioblastoma therapy.

Both cell lines were cultured in Dulbecco’s Modified Eagle’s Medium (DMEM; Gibco, Thermo Fisher Scientific, Waltham, MA, USA) supplemented with 10% fetal bovine serum (FBS), 1% penicillin–streptomycin, and 1% L-glutamine. Cells were maintained at 37 °C in a humidified incubator containing 5% CO_2_ under standard cell culture conditions [13]. The culture medium was replaced every two days, and cell morphology and confluency were routinely monitored using an inverted phase-contrast microscope to ensure optimal growth conditions and to exclude potential contamination.

For experimental procedures, cells were seeded into 96-well plates at a density allowing approximately 80–90% confluency after 24 h of incubation. Following cell attachment, the culture medium was replaced with fresh medium containing the indicated concentrations of melatonin and/or temozolomide according to the experimental design described below. All experiments were independently repeated three times. In each independent biological experiment, every treatment condition was analyzed in three technical replicate wells.

### 2.1. Cell Viability Assay (MTT)

Relative MTT metabolic activity was assessed using the MTT Cell Viability Assay Kit (Abcam, Cambridge, UK) according to the manufacturer’s instructions with minor modifications. The MTT assay is based on the reduction of the tetrazolium salt MTT by mitochondrial dehydrogenases in metabolically active cells, resulting in the formation of insoluble purple formazan crystals. Because MTT reflects cellular metabolic activity rather than direct viable-cell enumeration, results are reported as relative MTT metabolic activity (% of untreated control) [14].

Prior to the assay, all reagents and materials were equilibrated to room temperature and gently mixed. Following the treatment period, the culture medium was carefully removed from the wells. For adherent cells, the medium was aspirated directly from the wells to avoid disturbing the cell monolayer. Subsequently, 50 µL of serum-free culture medium and 50 µL of MTT reagent were added to each well. Background control wells containing MTT reagent and culture medium without cells were included to correct for non-specific absorbance.

The plates were incubated at 37 °C for 3 h to allow the formation of formazan crystals in viable cells. After incubation, 150 µL of MTT solvent was added to each well to dissolve the formazan crystals. The plates were protected from light and gently shaken on an orbital shaker for 15 min to ensure complete solubilization of the crystals.

Absorbance was measured at 590 nm using a microplate reader. Relative MTT metabolic activity was calculated as a percentage of the matched untreated control using the following formula:

Relative MTT metabolic activity (% of control) = (Absorbance of treated cells/Absorbance of corresponding untreated control cells) × 100(1)

For each independent biological experiment, treatment-associated absorbance values were normalized to the corresponding untreated control from the same experiment, which was defined as 100%. Technical replicate wells were first averaged within each biological experiment, and normalization was subsequently performed using the matched untreated control value. All normalized percentage values used for the melatonin-only and TMZ–melatonin experiments, including those presented in Figure 1 and Figure 2, were cross-checked against the corresponding original absorbance measurements to ensure consistency in the normalization procedure.

Preliminary dose–response experiments were performed to evaluate the effects of melatonin alone at concentrations of 100, 500, and 1000 µM in both U87MG and HUVEC cell lines.

### 2.2. Melatonin and Temozolomide Treatments

Temozolomide (TMZ; Sigma-Aldrich, St. Louis, MO, USA) was dissolved in dimethyl sulfoxide (DMSO) to prepare a stock solution and further diluted in culture medium to obtain the desired working concentrations. Melatonin (Sigma-Aldrich, St. Louis, MO, USA) was similarly dissolved in DMSO to prepare a stock solution and diluted in culture medium prior to treatment. Stock solutions were freshly prepared or stored at −20 °C and protected from light until use. The final concentration of DMSO in all experimental conditions did not exceed 0.1% (*v*/*v*). Control cells received an equivalent concentration of DMSO to exclude solvent-related effects. Cells were seeded at a density of 1 × 10^4^ cells/well in 96-well plates.

For single-agent experiments, cells were treated with melatonin at concentrations of 100, 500, and 1000 µM. Melatonin has been reported to exert anti-proliferative, antioxidant, and oncostatic effects in various cancer models, including glioblastoma [8,9,10].

For combination experiments, cells were exposed to increasing concentrations of temozolomide (TMZ; 1, 5, 10, 50, and 100 µM) either alone or in combination with 500 µM melatonin. Temozolomide is an oral alkylating agent and represents the current standard chemotherapeutic drug used in glioblastoma treatment due to its ability to cross the blood–brain barrier and induce DNA damage in tumor cells [1,2,3].

All treatments were applied after a 24 h cell adhesion period. Cells were exposed to melatonin and/or TMZ for 48 h before subsequent analyses. Untreated cells cultured under identical conditions served as the control group. Each treatment condition was analyzed in three technical replicate wells within each of three independent biological experiments.

### 2.3. Cell Cycle Analysis

Cell cycle distribution was analyzed by flow cytometry to evaluate the effects of melatonin and temozolomide treatments on cell cycle progression. Following treatment, cells were harvested, washed twice with phosphate-buffered saline (PBS), and fixed in cold 70% ethanol at 4 °C. Ethanol fixation allows permeabilization of the cell membrane and preservation of nuclear DNA for subsequent staining [15,16].

After fixation, cells were stained using a commercially available cell cycle analysis kit (NutriCulture, Ecotec, Erzurum, Türkiye) according to the manufacturer’s instructions. The staining solution contained propidium iodide (PI), which intercalates into DNA, enabling quantification of cellular DNA content by fluorescence intensity [15,16].

DNA content was analyzed to determine the distribution of cells in the G_0_/G_1_, S, and G_2_/M phases of the cell cycle. Flow cytometric analysis was performed using a BD Accuri C6 Flow Cytometer (BD Biosciences, San Jose, CA, USA). At least 10,000 events were acquired for each sample. Data were analyzed using FlowJo software (version 10.8), and the percentage of cells in each cell cycle phase was calculated using appropriate gating strategies [15,16].

### 2.4. Global DNA Methylation Analysis

Genomic DNA was isolated from U87MG and HUVECs using the DNeasy Blood and Tissue Kit (Qiagen, Hilden, Germany) according to the manufacturer’s protocol. The concentration and purity of the extracted DNA were determined using a NanoDrop 2000c spectrophotometer (Thermo Fisher Scientific, Waltham, MA, USA).

Global DNA methylation levels were assessed using the MethylFlash™ Methylated DNA 5-mC Quantification Kit (Colorimetric) (Epigentek Group Inc., Farmingdale, NY, USA; Cat. No. P-1034-96) according to the manufacturer’s instructions. This assay enables colorimetric quantification of global DNA methylation by specifically detecting 5-methylcytosine (5-mC) in an ELISA-like microplate format [17].

To evaluate the potential epigenetic effects of melatonin and temozolomide treatments, global DNA methylation levels were assessed in treated U87MG and HUVECs. For each reaction well, 20–200 ng of genomic DNA was used, with 100 ng per reaction considered optimal. DNA samples were diluted in nuclease-free water or TE buffer and added directly to the assay plate.

Following the binding and detection steps, absorbance was measured at 450 nm using a microplate reader. Global DNA methylation levels were calculated based on standard curves generated using the methylated DNA standards provided with the kit. Results were expressed as relative 5-methylcytosine (5-mC) content compared with untreated control samples. All samples were analyzed in triplicate, and blank controls were included in each assay to ensure accuracy and reproducibility.

### 2.5. Measurement of Cortistatin (CORT) Levels in Cell Lysates by Enzyme-Linked Immunosorbent Assay (ELISA)

Cortistatin levels were measured using a commercially available Human Cortistatin (CORT) ELISA Kit (Nepenthe, Kocaeli TUBITAK MAM, Gebze, Türkiye; Cat. No. NE010512101) according to the manufacturer’s instructions. ELISA is a widely used immunological technique for the quantitative detection of specific proteins based on antigen–antibody interactions and enzyme-mediated colorimetric or fluorometric signal generation [18].

Human U87MG and HUVECs were cultured under standard conditions as described above. Following the experimental treatments, cells were harvested and lysed for protein extraction. Briefly, adherent cells were detached using trypsin, collected by centrifugation, and washed three times with phosphate-buffered saline (PBS) to remove residual culture medium.

The resulting cell pellets were resuspended in PBS and subjected to three freeze–thaw cycles at −20 °C with gentle mixing to ensure efficient disruption of cellular membranes and release of intracellular proteins. The lysates were subsequently centrifuged at 1000× *g* for 15 min at 4 °C to remove cellular debris. The supernatants containing total cellular proteins were carefully collected and either immediately analyzed or stored at −80 °C until further use.

For ELISA, 100 µL of standards or cell lysate samples were added in triplicate to the antibody-precoated wells of the microplate, while blank control wells received PBS only. For cell lysate samples, 10 µL of balance solution was added to each well and mixed thoroughly, followed by the addition of 50 µL of enzyme conjugate to all wells except the blank controls. The plates were gently mixed, covered, and incubated for 1 h at 37 °C.

Subsequent washing steps, substrate reactions, and absorbance measurements at 450 nm were performed according to the manufacturer’s protocol. Because total protein content or viable cell number was not measured for lysate-input normalization, CORT findings are reported in the present study as exploratory ELISA-derived absorbance signals rather than as normalized intracellular concentrations. All measurements were performed in triplicate, and experiments were repeated independently to ensure reproducibility.

### 2.6. Statistical Analysis

All experiments were performed in three independent biological experiments conducted on separate experimental days using independently cultured cell preparations (*n* = 3 biological replicates). Within each biological experiment, each experimental condition was assessed in three technical replicate wells. Technical replicates were not considered independent biological observations; instead, the technical replicate measurements were averaged within each biological experiment to obtain one value per condition for each independent experiment.

For the melatonin-only experiments, linear mixed-effects models were fitted separately for HUVEC and U87MG cells. Melatonin concentration was included as a categorical fixed effect, whereas biological experiment was included as a random intercept to account for repeated measurements originating from the same independent experiment.

For the TMZ–melatonin experiments, linear mixed-effects models were fitted separately for HUVEC and U87MG cells and for each experimental outcome. TMZ concentration (1, 5, 10, 50, and 100 µM; categorical), melatonin exposure (absent or 500 µM), and the TMZ × melatonin interaction were included as fixed effects. Biological experiment was included as a random intercept. The TMZ × melatonin interaction term was used to formally assess whether the effect associated with melatonin co-treatment differed across TMZ concentrations.

Estimated marginal means were derived from the fitted models. Prespecified contrasts were used to compare TMZ alone with TMZ + 500 µM melatonin at each TMZ concentration and, for the melatonin-only experiments, to compare the tested melatonin concentrations. Multiplicity across the prespecified pairwise comparisons was controlled using the Holm method. Results are reported using model-derived effect estimates, 95% confidence intervals (CIs), and exact Holm-adjusted *p*-values. An adjusted *p*-value < 0.05 was considered statistically significant [19].

Data are presented as mean ± standard deviation (SD) of the three independent biological experiments unless otherwise stated. Because only three independent biological experiments were available, formal normality testing was not considered reliable; model adequacy was instead evaluated using residual diagnostics. Given the limited number of independent biological replicates, all model-based inferential findings were interpreted cautiously and are considered exploratory and hypothesis-generating [19].

## 3. Results

### 3.1. Effects of Melatonin and Temozolomide on Relative MTT Metabolic Activity in HUVEC and U87MG Cells

The effects of melatonin alone on relative MTT metabolic activity were evaluated in HUVEC and U87MG cells. For each independent biological experiment, treatment values were normalized to the matched untreated control, which was defined as 100%. In HUVECs, untreated control absorbance values were 0.092, 0.098, and 0.095 (mean 0.0950 ± 0.0030). Relative MTT metabolic activity was 85.36 ± 7.15%, 131.37 ± 8.75%, and 105.73 ± 6.79% following treatment with 100, 500, and 1000 µM melatonin, respectively. The highest relative MTT metabolic activity was observed at 500 µM melatonin.

In U87MG cells, untreated control absorbance values were 0.0655, 0.0725, and 0.0684 (mean 0.0688 ± 0.0035). Relative MTT metabolic activity was 103.80 ± 5.47%, 71.22 ± 1.63%, and 99.35 ± 1.48% following treatment with 100, 500, and 1000 µM melatonin, respectively. The lowest relative MTT metabolic activity was observed at 500 µM melatonin. Based on the contrasting response pattern observed in HUVEC and U87MG cells, 500 µM melatonin was selected for the subsequent TMZ combination experiments (Figure 1).

Data are presented as mean ± SD of three independent biological experiments (*n* = 3). Within each independent experiment, each treatment condition was analyzed in technical triplicate, and technical replicate measurements were averaged before normalization and statistical analysis.

The combined effects of TMZ and melatonin were subsequently assessed using relative MTT metabolic activity. In HUVECs, relative MTT metabolic activity remained comparatively high across the tested TMZ concentrations. Addition of 500 µM melatonin was associated with only modest differences at TMZ concentrations of 1–50 µM, whereas a more pronounced decrease was observed at 100 µM TMZ, from 194.67 ± 15.01% with TMZ alone to 154.00 ± 2.00% with TMZ plus melatonin. In U87MG cells, TMZ alone produced a non-linear response across the tested concentrations, with lower relative MTT metabolic activity observed particularly at 5 and 100 µM TMZ. Addition of 500 µM melatonin was associated with a different response pattern across TMZ concentrations. Relative MTT metabolic activity in the combination groups ranged from 90.33 ± 2.52% to 116.00 ± 5.29%, compared with 80.33 ± 7.57% to 157.33 ± 5.51% in the TMZ-alone groups.

Linear mixed-effects analysis demonstrated a significant TMZ × melatonin interaction for relative MTT metabolic activity in both HUVECs (F(4,18) = 7.28, *p* = 0.00113) and U87MG cells (F(4,18) = 16.33, *p* = 8.24 × 10^−6^), indicating that the magnitude and direction of the melatonin-associated effect differed across TMZ concentrations. In HUVECs, the only dose-specific contrast that remained significant after Holm correction was observed at 100 µM TMZ, where melatonin co-treatment was associated with an estimated −40.67 percentage-point difference relative to TMZ alone (95% CI: −54.58 to −26.76; Holm-adjusted *p* = 4.21 × 10^−5^). In U87MG cells, a significant difference was observed at 1 µM TMZ (estimated difference: −41.33 percentage points, 95% CI: −52.12 to −30.54; Holm-adjusted *p* = 1.13 × 10^−6^), whereas the contrasts at 5, 10, 50, and 100 µM TMZ were not significant after multiplicity adjustment.

Overall, HUVEC and U87MG cells exhibited distinct patterns of relative MTT metabolic activity in response to TMZ alone and TMZ–melatonin co-treatment. The significant interaction indicates that the melatonin-associated response depended on TMZ concentration; however, the present findings are exploratory and should not be interpreted as evidence of uniform pharmacological synergy, therapeutic selectivity, or a causal drug-interaction mechanism (Figure 1 and Figure 2).

### 3.2. Cell Cycle Distribution

Cell cycle analysis was performed to evaluate whether melatonin, TMZ, or their combined treatment altered cell cycle progression in HUVEC and U87MG cells. In HUVECs, no apparent treatment-associated shift was observed in the G_0_/G_1_, S, or G_2_/M populations under the tested conditions. Therefore, these data support the interpretation that endothelial cell cycle distribution was largely preserved during TMZ and melatonin exposure.

In U87MG cells, flow cytometric cell cycle analysis demonstrated that treatment with 100 µM TMZ resulted in a cell cycle distribution characterized by 51.52% of cells in the G_0_/G_1_ phase, 18.08% in the S phase, and 30.39% in the G_2_/M phase (Figure 3A). Following treatment with 500 µM melatonin, the proportion of cells in the G_0_/G_1_ phase increased to 56.46%, whereas the percentages of cells in the S and G_2_/M phases decreased to 17.04% and 27.38%, respectively (Figure 3B). Combined treatment with TMZ and melatonin further increased the G_0_/G_1_ population to 57.94%, accompanied by reductions in the S phase to 16.01% and in the G_2_/M phase to 26.76% (Figure 3C).

Overall, in U87MG cells, combined TMZ and melatonin treatment produced a modest increase in the G_0_/G_1_ population and a concomitant reduction in the S-phase fraction compared with TMZ alone. These findings suggest reduced proliferative progression rather than a definitive cell cycle arrest.

### 3.3. Global DNA Methylation-Related Assay Signals Following TMZ and Melatonin Treatments

Global DNA methylation-related assay signals were evaluated in HUVEC and U87MG cells following treatment with TMZ alone and in combination with 500 µM melatonin. The results below are presented as mean absorbance values from three independent biological experiments; variability is shown as SD in Figure 4.

#### 3.3.1. HUVECs

In HUVECs treated with TMZ alone, mean absorbance values were 0.177, 0.158, 0.170, 0.189, and 0.172 at 1, 5, 10, 50, and 100 µM TMZ, respectively. Following co-treatment with 500 µM melatonin, the corresponding values were 0.181, 0.162, 0.125, 0.166, and 0.170. Compared with TMZ alone, melatonin co-treatment was associated with lower values, particularly at 10 and 50 µM TMZ, whereas relatively similar values were observed at 1, 5, and 100 µM TMZ.

#### 3.3.2. U87MG Cells

In U87MG cells treated with TMZ alone, mean absorbance values were 0.187, 0.187, 0.165, 0.142, and 0.153 at 1, 5, 10, 50, and 100 µM TMZ, respectively. In the corresponding TMZ + melatonin groups, the values were 0.163, 0.153, 0.139, 0.109, and 0.129. Melatonin co-treatment was associated with lower assay values than TMZ alone across all tested TMZ concentrations in U87MG cells, with the largest numerical difference observed at 50 µM TMZ. For quantitative interpretation, the corresponding global DNA methylation values derived from the 5-mC calibration curve are presented as %5-mC in Figure 4A–D.

Mixed-effects analysis also identified a significant TMZ × melatonin interaction for the global DNA methylation-related assay signal in both HUVECs (F(4,18) = 215.67, *p* = 6.11 × 10^−15^) and U87MG cells (F(4,18) = 13.12, *p* = 3.56 × 10^−5^). In HUVECs, melatonin co-treatment was associated with small increases in assay signal at 1 and 5 µM TMZ (estimated difference: +0.004 at both concentrations; 95% CI: 0.00095 to 0.00705; Holm-adjusted *p* = 0.0393), whereas lower assay signals were observed at 10 µM (estimated difference: −0.045, 95% CI: −0.0481 to −0.0419; adjusted *p* = 2.28 × 10^−16^) and 50 µM TMZ (estimated difference: −0.023, 95% CI: −0.0261 to −0.0199; adjusted *p* = 2.09 × 10^−11^). The difference at 100 µM TMZ was not statistically significant (adjusted *p* = 0.186). In U87MG cells, melatonin co-treatment was associated with lower assay signals at all tested TMZ concentrations, with estimated differences ranging from −0.024 to −0.034 and all Holm-adjusted *p*-values < 2 × 10^−12^. These inferential analyses were performed on background-corrected assay measurements, whereas calibration-derived %5-mC values are provided in Figure 4 for quantitative presentation of global DNA methylation.

Overall, HUVEC and U87MG cells exhibited distinct treatment-associated patterns in global DNA methylation-related assay signals following TMZ alone and TMZ–melatonin co-treatment. The corresponding calibration curve and derived %5-mC results are shown in Figure 4A–D. These observations are considered exploratory and do not, by themselves, establish a causal epigenetic mechanism.

### 3.4. Treatment-Associated Cortistatin Responses in HUVEC and U87MG Cells

CORT levels were quantified by ELISA in HUVEC endothelial cells and U87MG glioblastoma cells following treatment with melatonin alone, TMZ alone, and combined TMZ + melatonin treatment. Absorbance values were compared with untreated control groups for each cell line (HUVEC control: 0.397; U87MG control: 0.433).

#### 3.4.1. TMZ Single Treatment

TMZ treatment produced different CORT response patterns in HUVEC and U87MG cells. In HUVECs, TMZ did not induce a consistent increase in CORT levels across the tested dose range. CORT absorbance values were lower than control at 1 µM (0.317) and 5 µM (0.339), showed a modest increase at 10 µM (0.444), remained close to control at 50 µM (0.405), and decreased again at 100 µM TMZ (0.370). In contrast, U87MG cells showed more evident dose-dependent alterations. CORT levels were reduced at 1 µM TMZ (0.286), approached control values at 5 µM (0.396), increased at intermediate concentrations, particularly at 10 µM (0.557) and 50 µM TMZ (0.521), and decreased again at 100 µM TMZ (0.401).

#### 3.4.2. Melatonin Single Treatment

Melatonin alone produced limited changes in CORT levels in HUVECs but a more pronounced response in U87MG cells. In HUVECs, CORT absorbance values remained close to control at 100 µM (0.382) and 500 µM melatonin (0.397), whereas a decrease was observed at 1000 µM melatonin (0.334). By contrast, U87MG cells exhibited increased CORT levels following melatonin treatment, with absorbance values rising at 100 µM (0.588), reaching the highest level at 500 µM (0.983), and remaining elevated at 1000 µM (0.566), although lower than the value observed at 500 µM.

#### 3.4.3. Combined TMZ + Melatonin Treatment

Combined TMZ + melatonin treatment showed distinct cell-type-dependent patterns in relative CORT signals. In HUVECs, co-treatment resulted in relatively limited changes compared with the corresponding untreated control. Relative CORT levels were approximately 112% of control at 1 µM TMZ + melatonin, 98% at 5 µM, 98% at 10 µM, 81% at 50 µM, and 101% at 100 µM TMZ + melatonin, indicating an overall relatively stable response across most treatment conditions.

In U87MG cells, a more pronounced treatment-associated pattern was observed. Relative CORT levels increased to approximately 134% of control at 5 µM TMZ + melatonin, reached the highest relative signal at 10 µM TMZ + melatonin (approximately 337% of control), and remained elevated at 50 µM TMZ + melatonin (approximately 242% of control). At 100 µM TMZ + melatonin, the relative CORT signal decreased to approximately 91% of control. Thus, the strongest relative CORT responses were observed at intermediate TMZ concentrations during melatonin co-treatment.

Overall, the semi-quantitative CORT analysis revealed distinct treatment-associated patterns between the two cell lines. HUVECs showed comparatively stable relative CORT signals, whereas U87MG cells exhibited substantially higher relative signals at intermediate TMZ concentrations under melatonin co-treatment (Figure 5). These findings should be interpreted as relative, semi-quantitative changes rather than absolute differences in intracellular CORT concentration.

## 4. Discussion

The present study examined treatment-associated differences in relative MTT metabolic activity, cell-cycle distribution, global DNA methylation-related assay signals, and CORT measurements in U87MG glioblastoma and HUVEC endothelial cells exposed to melatonin and TMZ. The two cell types exhibited different response patterns across these endpoints. Because the study used a single glioblastoma cell line, three independent biological experiments, global rather than locus-specific methylation measurements, and no functional manipulation of CORT signaling, the findings are interpreted as exploratory associations rather than evidence of tumor selectivity or a defined epigenetic or stress-response mechanism.

### 4.1. Differential Cellular Responses to Melatonin and TMZ

The results showed different MTT metabolic response patterns in endothelial and glioblastoma cells. In HUVECs, melatonin alone produced concentration-dependent changes in relative MTT metabolic activity, with the highest value observed at 500 µM. Because MTT is a metabolic assay, values above the untreated control should not be interpreted as direct evidence of increased viable-cell number or hyper-proliferation.

In U87MG cells, 500 µM melatonin produced the lowest relative MTT metabolic activity among the tested melatonin concentrations. This concentration was therefore selected for the combination experiments as an exploratory condition that produced contrasting MTT response patterns in U87MG and HUVECs.

During TMZ co-treatment, the direction and magnitude of the melatonin-associated difference varied across TMZ concentrations and differed between HUVEC and U87MG cells. These data support the presence of treatment-condition- and cell-type-associated response patterns but do not establish selective tumor targeting, cytoprotection of normal tissue, or pharmacological synergy.

### 4.2. Cell Cycle Modulation Induced by Melatonin and Temozolomide

Cell cycle regulation is closely associated with glioblastoma cell proliferation and response to chemotherapeutic stress. Temozolomide exerts its antitumor effect mainly by inducing DNA alkylation damage, which may activate DNA damage response pathways and cell cycle checkpoints in glioblastoma cells [3,15]. In the present study, HUVECs did not show a meaningful alteration in cell cycle distribution following melatonin, TMZ, or combined treatment. This finding is consistent with the preserved viability and relatively stable global DNA methylation profile observed in HUVECs, suggesting that endothelial cell cycle homeostasis was largely maintained under the applied experimental conditions.

In contrast, U87MG glioblastoma cells showed a moderate treatment-associated shift in cell cycle distribution. TMZ treatment resulted in 51.52% of cells in the G_0_/G_1_ phase, 18.08% in the S phase, and 30.39% in the G_2_/M phase. Melatonin treatment increased the G_0_/G_1_ population to 56.46%, while the combined TMZ and melatonin treatment further increased this fraction to 57.94%. In parallel, the S phase population decreased from 18.08% in the TMZ-treated group to 16.01% in the combined treatment group. These findings suggest that melatonin, particularly in combination with TMZ, may contribute to a modest restriction of cell cycle progression in U87MG cells.

Previous studies have reported that melatonin may regulate cancer cell proliferation by modulating cyclins, cyclin-dependent kinases, oxidative stress responses, mitochondrial function, and PI3K/AKT-related survival pathways [8,9,10,11,20]. In glioblastoma and neuroblastoma models, melatonin has also been shown to enhance TMZ-induced antitumor responses, including apoptosis and cell cycle-related changes [11]. Therefore, the increased G_0_/G_1_ fraction and reduced S-phase population observed in the combined treatment group may reflect a reduction in proliferative progression rather than a strong or complete modest G_0_/G_1_ accumulation.

Overall, the cell-cycle findings indicate modest treatment-associated differences in U87MG cells, including a higher G_0_/G_1_ fraction and lower S-phase fraction in the combined-treatment condition. These observations are compatible with reduced proliferative progression but do not establish a specific cell-cycle mechanism. The lack of a pronounced shift in HUVECs further indicates that the response patterns differed between the two cell types under the tested conditions.

### 4.3. Treatment-Associated Changes in Global DNA Methylation-Related Measurements

Global DNA methylation-related assay signals showed distinct treatment-associated patterns in HUVEC and U87MG cells. The mixed-effects analysis demonstrated a significant TMZ × melatonin interaction in both cell types, indicating that the effect associated with melatonin co-treatment depended on TMZ concentration rather than representing a uniform response across the tested dose range.

In HUVECs, the direction of the melatonin-associated difference varied according to TMZ concentration. Small increases in assay signal were observed at the lowest TMZ concentrations, whereas lower values were detected at 10 and 50 µM TMZ. No significant difference was observed at 100 µM. This non-uniform pattern does not support a simple dose-dependent effect of melatonin on global DNA methylation-related measurements in endothelial cells.

In contrast, U87MG cells showed consistently lower assay signals following melatonin co-treatment across all tested TMZ concentrations. Although this pattern suggests that the combined treatment was associated with a reproducible difference in the global methylation-related endpoint in U87MG cells, the present data do not establish that melatonin directly induces DNA hypomethylation or epigenetic remodeling.

Epigenetic dysregulation is an important feature of glioblastoma progression and therapeutic resistance [4,5,6,7]. Previous experimental studies have also suggested that TMZ and melatonin may influence epigenetic processes in cancer models [9,10,12]. However, the present study assessed a global DNA methylation-related endpoint and did not investigate locus-specific methylation, DNA methyltransferase activity, ten-eleven translocation enzymes, chromatin modifications, or downstream gene-expression changes. Therefore, the observed treatment-associated differences should be interpreted as descriptive and hypothesis-generating rather than as evidence of a defined epigenetic mechanism.

The different response patterns observed between HUVEC and U87MG cells may reflect intrinsic differences in cellular context; however, this comparison should not be interpreted as evidence of tumor-specific or neural-selective epigenetic effects. HUVECs represent a non-malignant endothelial comparator and not a non-malignant neural control. Further studies using normal astrocytes or other relevant neural cell models, together with locus-specific methylation and epigenetic enzyme analyses, are required to determine the biological and functional significance of these observations.

### 4.4. Treatment-Associated CORT Responses in HUVEC and U87MG Cells

CORT is a neuropeptide structurally related to somatostatin and has been implicated in inflammatory and proliferative regulation [21,22]. In the present study, CORT measurements showed different treatment-associated patterns in HUVEC and U87MG cells. These observations are exploratory and do not establish CORT as a functional mediator of the response to TMZ or melatonin.

HUVECs showed comparatively limited variation in CORT assay values across most treatment conditions, whereas U87MG cells showed larger numerical changes under several conditions. These between-cell-line differences should be interpreted as descriptive because the present experiments did not manipulate CORT signaling or assess downstream receptor-dependent responses.

In U87MG cells, higher CORT assay values were observed at intermediate TMZ concentrations during melatonin co-treatment, particularly at 10 and 50 µM TMZ. Although this pattern may be relevant to treatment-associated cellular responses, it does not demonstrate activation of a defined stress-response pathway.

The CORT pattern occurred alongside differences in MTT metabolic activity, cell-cycle distribution, and global DNA methylation-related measurements. However, temporal or causal relationships among these endpoints were not tested. It therefore remains unknown whether CORT changes are upstream mediators, downstream consequences, or independent correlates of treatment exposure. Taken together, the CORT data should be regarded as an exploratory treatment-associated observation. Functional studies of CORT receptors and downstream signaling, together with appropriate normalization of lysate input, are required before CORT can be considered a mechanistic or stress-response biomarker in this experimental context.

### 4.5. Integrated Interpretation of Treatment-Associated Responses

Considered together, the MTT, cell-cycle, global DNA methylation-related, and CORT measurements show that U87MG and HUVECs respond differently across TMZ and melatonin treatment conditions. The convergence of several endpoints may help generate hypotheses regarding treatment-associated cellular responses, but it does not establish an integrated causal mechanism.

In U87MG cells, combined treatment was associated with differences in relative MTT metabolic activity, modest shifts in cell-cycle distribution, lower global DNA methylation-related assay signals under several conditions, and changes in CORT measurements. Each endpoint was assessed independently, and the present design does not demonstrate that one of these changes caused another. Accordingly, lower global methylation-related assay signals should not be interpreted as proof of epigenetic remodeling, and higher CORT measurements should not be interpreted as proof of stress-responsive neuropeptide signaling. Both observations require targeted mechanistic validation.

HUVECs showed a different overall response profile from U87MG cells, but this comparison is limited to an endothelial non-malignant model. It cannot establish neural safety or tumor-specific selectivity. The differences observed between U87MG and HUVECs therefore support only a cell-context-dependent description of the measured responses. Additional glioblastoma lines, patient-derived models, and non-malignant neural controls are required to determine the reproducibility and biological relevance of these findings. Collectively, the present data are best interpreted as preliminary evidence of treatment-condition- and cell-type-associated differences across several cellular readouts. They provide a basis for future mechanistic studies but do not demonstrate synergy, therapeutic selectivity, epigenetic reprogramming, or a CORT-mediated stress-response pathway.

### 4.6. Clinical and Translational Implications

Glioblastoma remains one of the most aggressive and treatment-resistant primary brain tumors, and resistance to temozolomide represents a major obstacle in current therapeutic strategies. Increasing evidence indicates that tumor heterogeneity and epigenetic dysregulation contribute substantially to therapeutic failure in glioblastoma patients [4,5,6]. Therefore, identifying agents capable of modulating the tumor response to TMZ without increasing toxicity to healthy tissues represents an important translational goal.

The present findings are insufficient to establish melatonin as an adjunct therapeutic strategy. Rather, they identify experimental response patterns that may justify further investigation of melatonin–TMZ combinations in additional glioblastoma models and in non-malignant neural controls.

Although melatonin has been reported to influence cellular signaling, mitochondrial function, and epigenetic regulation in cancer models [11], the current study did not directly measure these pathways. The global DNA methylation-related observations should therefore not be used to infer a specific epigenetic mechanism of TMZ responsiveness.

Similarly, the treatment-associated CORT pattern observed in U87MG cells requires confirmation using quantitatively normalized CORT measurements and functional pathway studies before neuroendocrine or stress-signaling implications can be proposed. Thus, the translational significance of the present findings remains uncertain. The results should be considered hypothesis-generating and require independent replication, mechanistic validation, and evaluation in models that more closely represent the neural tumor microenvironment.

### 4.7. Study Limitations and Future Perspectives

Despite providing novel insights into the interaction between melatonin and temozolomide in glioblastoma cells, several limitations of the present study should be acknowledged. First, the experimental design was based on in vitro cell culture models using a single glioblastoma cell line. Although U87MG cells are widely used in glioblastoma research, glioblastoma is characterized by marked molecular and cellular heterogeneity; therefore, the present findings should be validated in additional glioblastoma cell lines and patient-derived models.

Second, a major limitation of this study is that global 5-methylcytosine quantification provides only a broad estimate of DNA methylation status and does not identify locus-specific methylation changes, epigenetic enzyme activity, or downstream transcriptional consequences. Therefore, the observed methylation changes should not be interpreted as definitive evidence of mechanistic epigenetic remodeling. Future studies should include DNA methyltransferase and ten-eleven translocation enzyme expression or activity analyses, histone modification profiling, gene-specific methylation assays, and transcriptomic approaches to clarify whether the observed global methylation changes translate into functionally meaningful epigenetic regulation.

Third, important analytical limitations should be considered when interpreting the CORT findings. Complete run-specific calibration curves were not available for all independent ELISA experiments; therefore, the CORT data should be interpreted as semi-quantitative relative measurements rather than as absolute concentrations. Accordingly, background-corrected OD values were normalized to the corresponding untreated control within each experimental run and are presented as relative CORT levels. In addition, although all cultures were initiated at the same seeding density and processed using identical lysate preparation volumes, CORT measurements were not normalized to total protein content or viable cell number. Therefore, differences in ELISA-derived CORT signals may partly reflect differences in the amount of cellular material between treatment conditions. The observed treatment-associated changes in CORT should consequently be regarded as exploratory relative signals rather than definitive quantitative evidence of altered intracellular CORT abundance. Moreover, the present study does not establish whether CORT functionally mediates TMZ–melatonin-associated cellular stress or represents a downstream consequence of this response. Future studies should incorporate complete run-specific calibration curves, normalization to total protein content or viable cell number, and functional analyses of CORT signaling pathways, receptor interactions, and downstream transcriptional responses.

Finally, the present study was conducted exclusively under in vitro conditions. Additional in vivo studies and preclinical models are required to determine whether the observed changes in global DNA methylation and relative CORT levels are maintained within the complex tumor microenvironment of glioblastoma.

Despite these limitations, the study identifies treatment-associated differences between U87MG and HUVECs across relative MTT metabolic activity, cell-cycle distribution, global DNA methylation-related measurements, and relative CORT responses. Further investigation using additional glioblastoma models, non-malignant neural controls, fully quantitative and appropriately normalized CORT measurements, and targeted mechanistic approaches is required to determine the functional relevance of these observations.

## 5. Conclusions

In conclusion, U87MG glioblastoma cells and HUVEC endothelial cells exhibited different response patterns following TMZ and melatonin exposure across relative MTT metabolic activity, cell-cycle distribution, global DNA methylation-related measurements, and CORT. These findings are preliminary and hypothesis-generating. The present data do not establish therapeutic selectivity, pharmacological synergy, a causal epigenetic mechanism, or a CORT-mediated stress-response pathway. Confirmation in additional glioblastoma models, non-malignant neural controls, and targeted mechanistic studies is required. CORT was semi-quantitatively assessed.

## Figures and Tables

**Figure 1:**
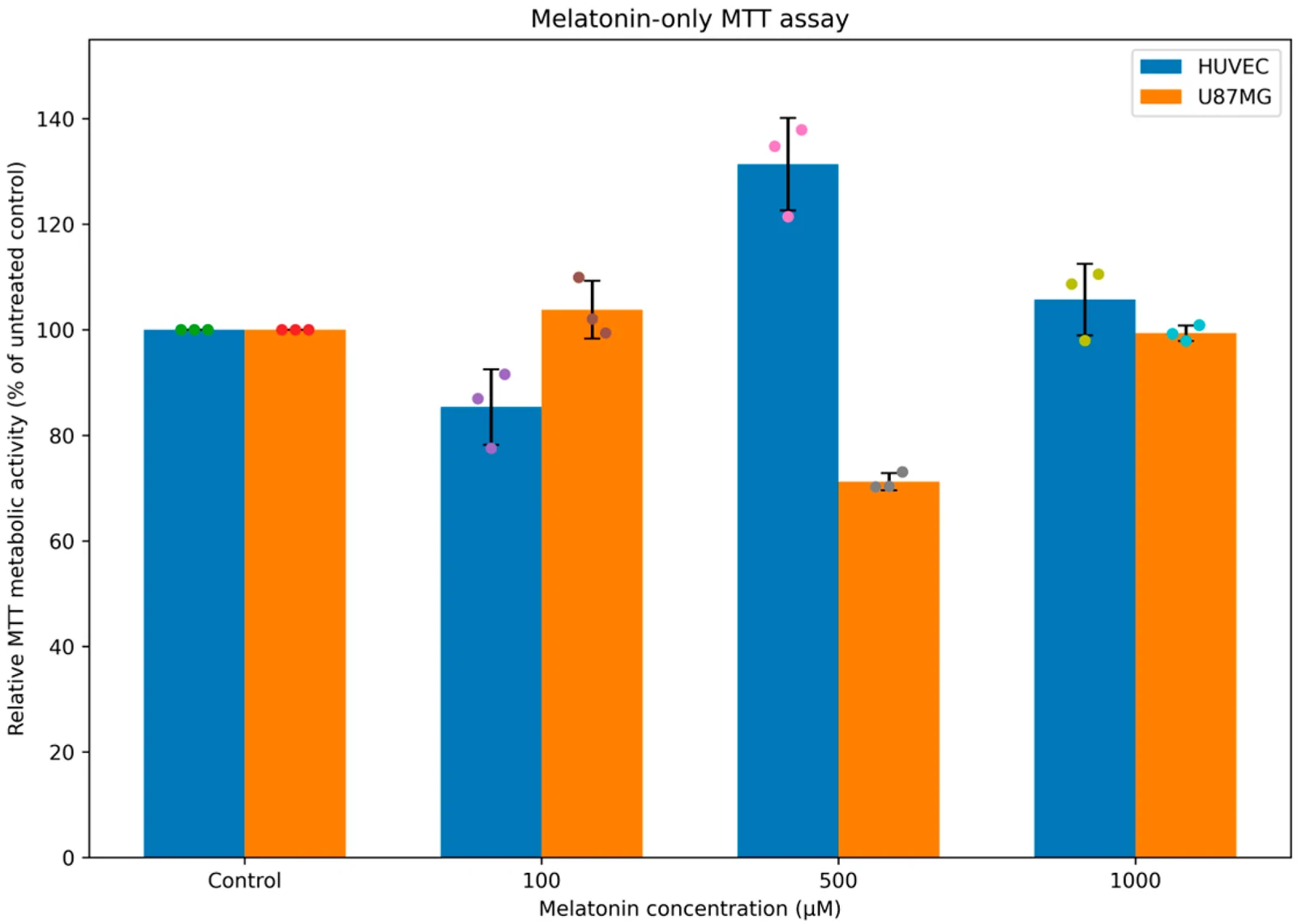
Effect of melatonin alone on relative MTT metabolic activity in HUVEC and U87MG cells. Colored circles represent the individual values obtained from the three independent biological experiments. Cells were treated with melatonin at 100, 500, and 1000 µM. MTT absorbance values for each treatment condition were normalized to the matched untreated control from the same independent biological experiment, which was defined as 100%. Data are presented as mean ± SD of three independent biological experiments (*n* = 3), with individual biological replicate values shown. Each biological experiment included three technical replicate wells, which were averaged before normalization and statistical analysis.

**Figure 2:**
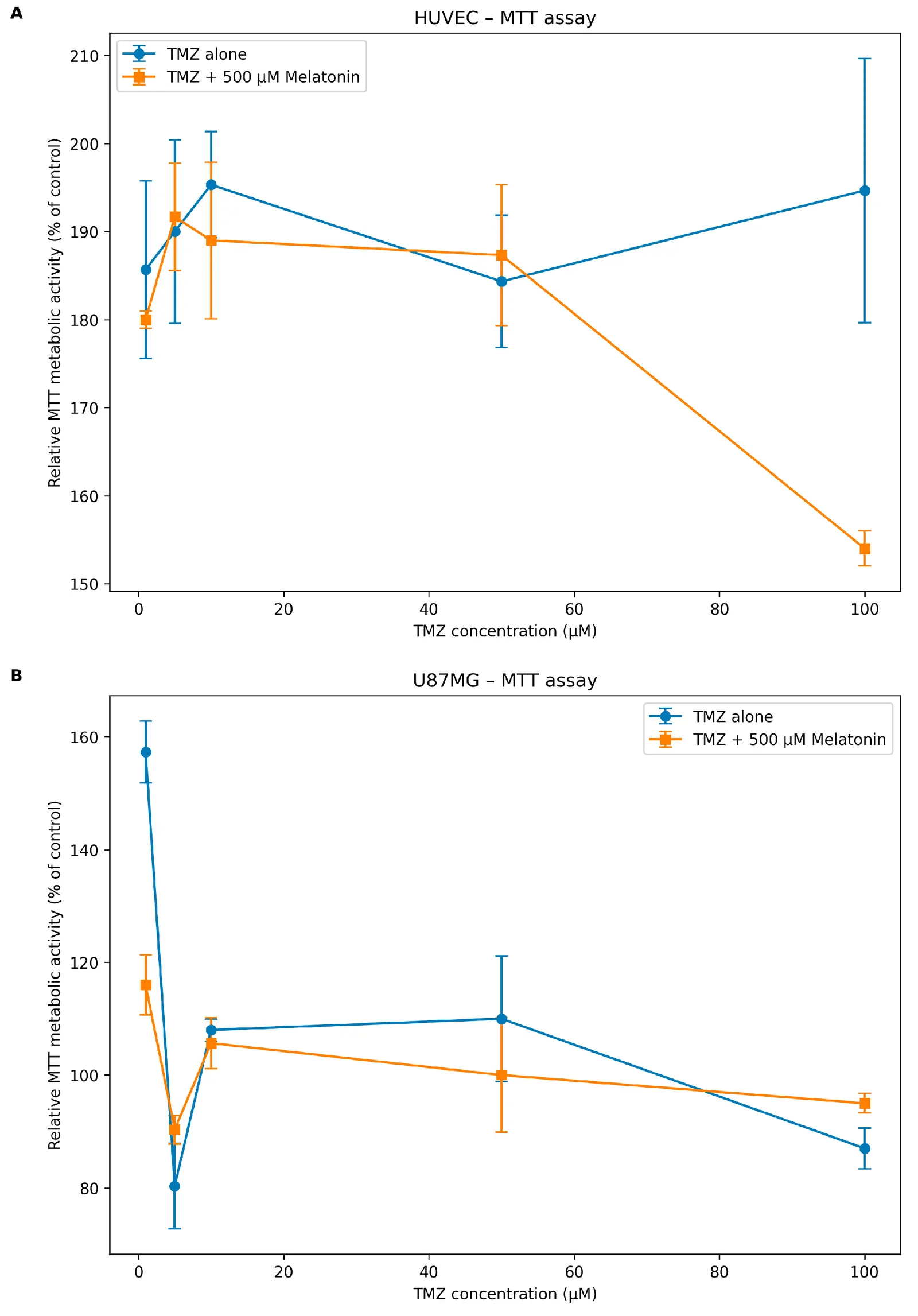
Effects of TMZ and melatonin co-treatment on relative MTT metabolic activity in HUVEC and U87MG cells. (**A**) Relative MTT metabolic activity of HUVECs following treatment with increasing concentrations of TMZ (1, 5, 10, 50, and 100 µM), either alone or in combination with 500 µM melatonin. (**B**) Relative MTT metabolic activity of U87MG glioblastoma cells following the same TMZ concentrations, either alone or in combination with 500 µM melatonin. Cells were exposed to the treatments for 48 h. MTT values are expressed relative to the corresponding untreated control from the same independent biological experiment. Data are presented as mean ± SD of three independent biological experiments (*n* = 3). Technical replicate measurements were averaged within each biological experiment before analysis. Error bars represent SD.

**Figure 3:**
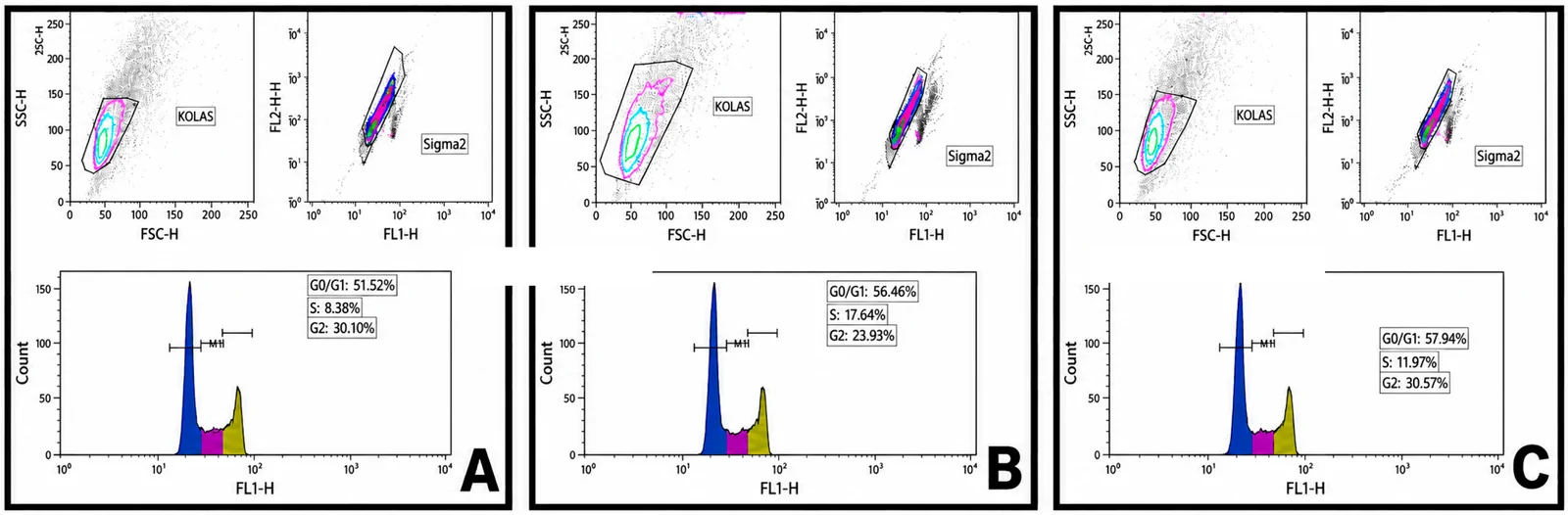
Effects of temozolomide, melatonin, and their combined treatment on cell cycle distribution in U87MG cells. In the DNA-content histograms, blue represents the G_0_/G_1_ phase, magenta represents the S phase, and yellow represents the G_2_/M phase. (**A**) TMZ treatment. (**B**) Melatonin treatment. (**C**) Combined TMZ + melatonin treatment. Cell cycle distribution was analyzed by flow cytometry in U87MG cells.

**Figure 4:**
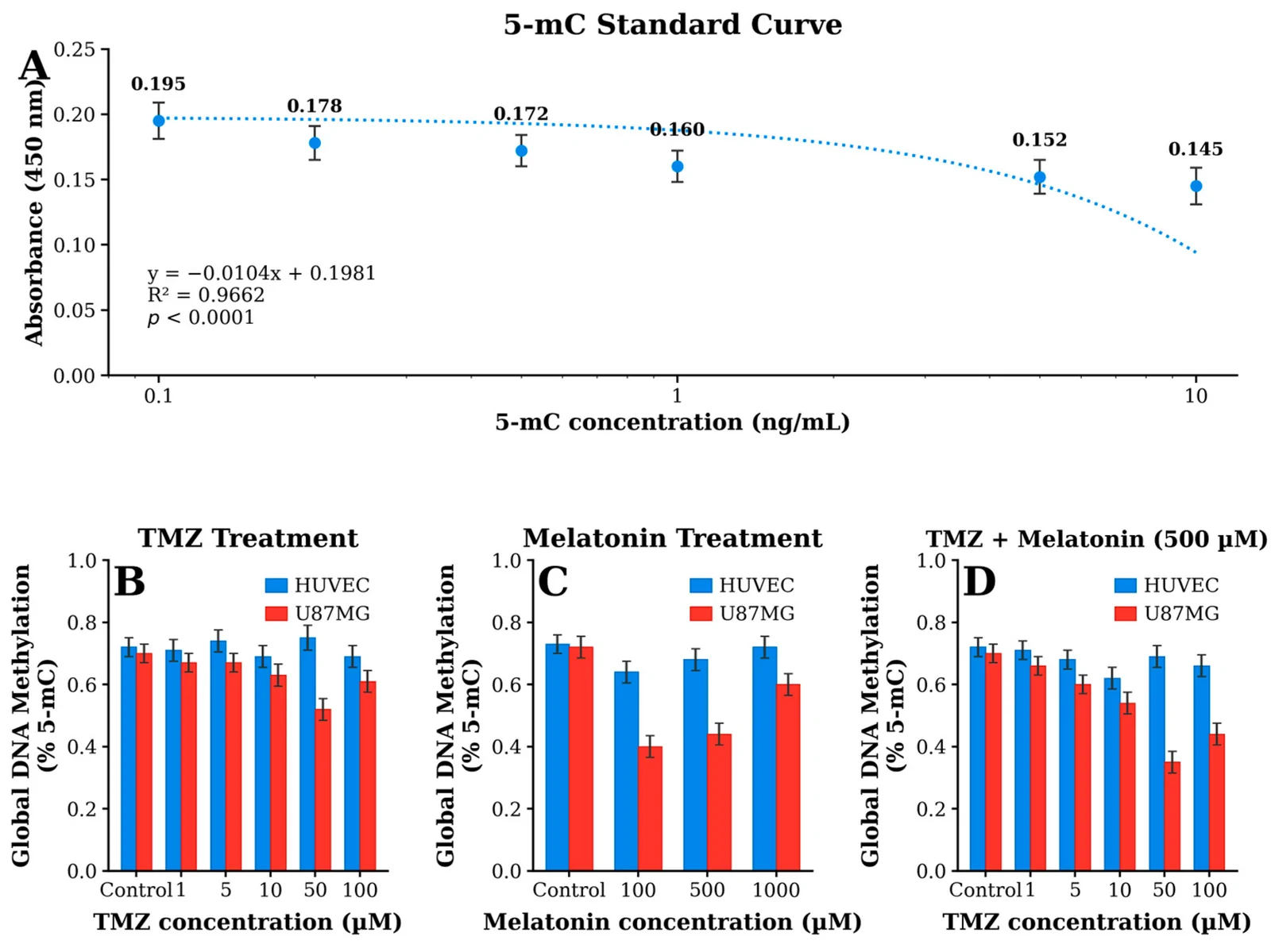
Global DNA methylation levels in HUVEC and U87MG cells under TMZ and melatonin treatment conditions. (**A**) 5-mC standard curve showing the relationship between 5-mC concentration and absorbance at 450 nm. (**B**) TMZ treatment. (**C**) Melatonin treatment. (**D**) Combined TMZ + melatonin (500 µM) treatment. Global DNA methylation-related assay signals were measured using a colorimetric 5-methylcytosine (5-mC) assay. Data are presented as mean ± SD.

**Figure 5:**
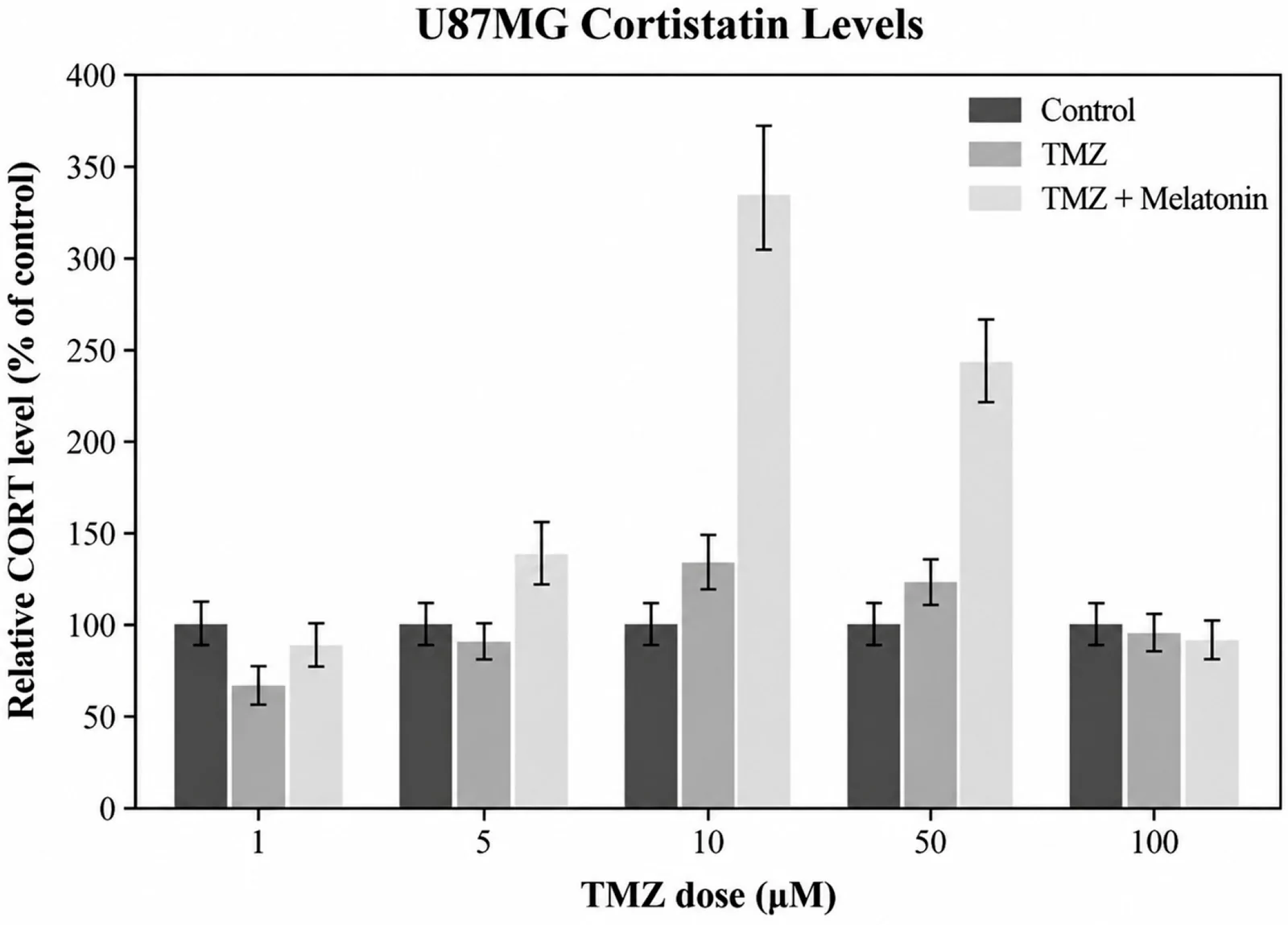
Complete run-specific calibration curves were not available for all independent ELISA runs; therefore, CORT data should be interpreted as semi-quantitative rather than as absolute concentrations.

## Data Availability

The original contributions presented in this study are included in the article. Further inquiries can be directed to the corresponding author.
